# 1514. Evaluating Missed Opportunities for Confirmatory HIV Testing at a Midwest Medical Center

**DOI:** 10.1093/ofid/ofad500.1349

**Published:** 2023-11-27

**Authors:** Lauryn E Caster, Harlan R Sayles, Paul D Fey, Sara H Bares

**Affiliations:** University of Nebraska Medical Center, Papillion, Nebraska; University of Nebraska Medical Center, Papillion, Nebraska; Univeristy of Nebraska Medical Center, Omaha, Nebraska; University of Nebraska Medical Center, Papillion, Nebraska

## Abstract

**Background:**

Current recommendations for HIV testing begin with a 4^th^ generation antigen/antibody (Ag/Ab) combination immunoassay followed by an antibody differentiation confirmatory assay. Discordant results may represent a false positive on the screening test or early HIV infection and must be followed up by a confirmatory HIV RNA test, but this is not done reflexively. As a result, there is a potential for missed opportunities for this confirmatory testing.

**Methods:**

We conducted a retrospective chart review of adult patients at a Midwest academic medical center with discordant results on the screeningHIV-1/2 Ag/Ab combination assay between Jan. 2017 and Feb. 2021. We assessed if appropriate confirmatory testing was performed, identified barriers to missed opportunities for confirmatory testing, and used Wilcoxon rank sum tests, chi-square tests, and Fisher’s exact tests to evaluate differences between those who did and did not receive confirmatory testing.

**Results:**

Of the 64 patients with a positive HIV antibody component of the Ag-Ab immunoassay and a negative confirmatory test, 35 (55%) patients completed a HIV RNA viral load. Of the 35 patients receiving the test, 3 (9%) patients tested positive for HIV. Younger age was significantly associated with completion of appropriate confirmatory testing (p=0.03), but other factors including sex, race, insurance status, having a primary care provider, specialty of ordering provider, encounter location, and test indication were not.

**Conclusion:**

Our review identified multiple missed opportunities for confirmatory HIV testing following a positive antibody component on the HIV screening test, with younger age being a significant factor in whether an RNA test was completed or not. Our results suggest that missed opportunities to diagnose patients with HIV is occurring over a heterogenous group of patients and providers. Therefore, systemic interventions are needed to address both provider and patient centered factors preventing early diagnosis of HIV. The findings of this study emphasize the need for reflexive HIV RNA viral loads on patients when the results of the HIV Ag-Ab immunoassay and confirmatory test are discordant.

**Disclosures:**

**Sara H. Bares, MD**, Gilead Sciences: Expert Testimony|GSK ViiV Healthcare: Grant/Research Support|Janssen: Grant/Research Support

